# CO_2_ Adsorption on Activated Carbon Honeycomb-Monoliths: A Comparison of Langmuir and Tóth Models

**DOI:** 10.3390/ijms13078388

**Published:** 2012-07-05

**Authors:** Diana P. Vargas, Liliana Giraldo, Juan C. Moreno-Piraján

**Affiliations:** 1 Department of Chemistry, Faculty of Sciences, National University of Colombia, Avenida Carrera 30 No. 45-03, Bogotá, Colombia; E-Mails: dpvargasd@unal.edu.co (D.P.V.); lgiraldogu@unal.edu.co (L.G.); 2 Department of Chemistry, Faculty of Sciences, Andes University, Carrera 1 No. 18 A 10, Bogotá, Colombia

**Keywords:** activated carbon, carbon monolith, chemical activation, CO_2_ adsorption, Langmuir, Tóth

## Abstract

Activated carbon honeycomb-monoliths with different textural properties were prepared by chemical activation of African palm shells with H_3_PO_4_, ZnCl_2_ and CaCl_2_ aqueous solutions of various concentrations. The adsorbents obtained were characterized by N_2_ adsorption at 77 K, and their carbon dioxide adsorption capacities were measured at 273 K and 1 Bar in volumetric adsorption equipment. The experimental adsorption isotherms were fitted to Langmuir and Tóth models, and a better fit was observed to Tóth equation with a correlation coefficient of 0.999. The maximum experimental values for adsorption capacity at the highest pressure (2.627–5.756 mmol·g^−1^) are between the calculated data in the two models.

## 1. Introduction

Adsorbent porous materials have become very important for adsorption-based processes in environmental and catalytic applications. The most used adsorbents are zeolites, pillared clays, mesoporous silica materials, activated carbons and more recently, Metal Organic Frameworks (MOFs) [1–6]. Carbon-based materials are well known, being widely applied at industrial scale as adsorbents [3,5–7], electrodes [8,9], fibers [9], nanotubes [10], monoliths [11,12], catalysts and/or and catalytic supports [4,11].

Among the numerous structural forms existing for activated carbons, carbon monoliths have received increasing interest in gas phase adsorption because of the low restrictions to mass transfer diffusion, the low pressure drop produced in packed beds, and their high mechanical and chemical resistance [13,14]. This is the main reason for their extended use in gas adsorption applications like methane, hydrogen, and carbon dioxide capture and storage [11–14], which are important issues in the energy and environmental scenario.

This work is aimed at CO_2_ adsorption on activated carbon monoliths at 273 K and 1 Bar, obtained from African palm shell by chemical activation with H_3_PO_4_, ZnCl_2_ and CaCl_2_ aqueous solutions of various concentrations. Among the existing theoretical adsorption models, Langmuir and Tóth models were selected to fit the experimental data of CO_2_ adsorption [15–17]. The Langmuir model is based on the assumption of a stronger adsorption energy for the first layer of adsorbed molecules, with a higher pressure being required for further adsorbed layers. Langmuir equilibrium equation can be expressed as [15–19]:

(1) $$q=\frac{q_{max}k_{L}P}{1+k_{L}P}$$

where *K_L_* is the Langmuir constant, related to the adsorption energy and the affinity of gas molecules to the adsorption sites, and *q_max_* the maximum adsorbed concentration (maximum adsorption capacity).

Tóth model is actually a modification of Langmuir model, which has the advantage of predicting the right adsorption limits when pressure approaches zero and infinite values, thus reducing deviation errors between experimental data and calculated values of adsorption equilibrium. This model is derived from the potential theory and assumes a quasi-Gaussian energy distribution in which most of the adsorption sites have a smaller adsorption energy than the peak adsorption energy. Tóth equation has three adjustable parameters, and is a useful tool to describe adsorption equilibrium on heterogeneous systems and multilayer adsorption. It can be expressed in the following way [15–18]:

(2) $$\frac{q}{q_{m}}=\frac{\alpha_{T}P}{{\left[1+{\left(\alpha_{T}P\right)}^{n}\right]}^{1/n}}$$

where *α_T_* and *q_m_* are two parameters similar to those in the Langmuir equation (*K_L_* and *q_max_*) and *n* is an exponent related to the heterogeneity of the adsorbent surface.

## 2. Results and Discussion

### 2.1. Preparation and Characterization of Materials

Preparation of monolith structures usually requires additional binding components to assure a better particle adhesion during pressure agglomeration for a higher mechanical resistance. However, the binding components can produce a significant decrease of porosity in the resulting material. For this reason, the preparation of carbon monoliths in this work was carried out without any binder material [20].

A usual procedure for preparing monolith adsorbents without binding materials is based on the use of an activating agent that can interact with the lignocellulosic precursor, producing dehydration reactions as well as degradation and condensation of the biopolymer molecules, becoming substances able to produce agglomeration of the particles during the pressure treatment of the solid [21].

The impregnation step was carried out by adding the granular solid precursor to calcium chloride, zinc chloride or phosphoric acid solutions. In these treatments with an excess of liquid solution, part of the organic products resulting from the chemical transformations and present in the solution can be concentrated by partial evaporation of the solvent during heating, resulting in some binding properties similar to a tarr product, and thus becoming a kind of binding component. In this way, the impregnated precursor materials show a slurry-like texture and a plastic behavior as a result of the chemical treatment which helps preparing the monoliths when applying pressure, resulting in a significant reduction of the interparticle space and as a consequence a remarkably consistent final material. The monolith structures thus obtained were stable during the following steps of thermal treatment and washing.

**Figure 1:**
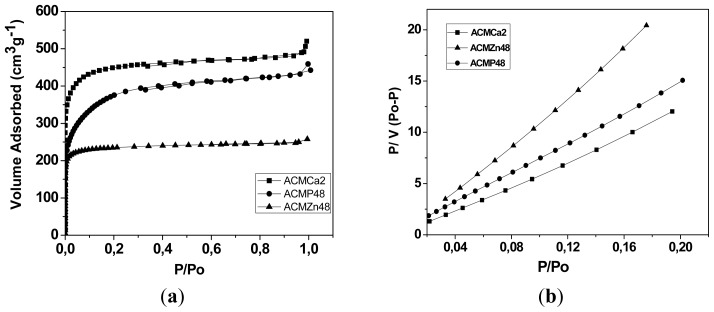
(**a**) Nitrogen adsorption isotherms at 77 K; (**b**) Data fitting to BET model. Selected samples are the best of each series according to their adsorption capacity.

All monoliths samples were characterized by nitrogen adsorption at 77 K. Figure 1 shows the adsorption isotherms corresponding to some of the samples, particularly those of highest adsorption capacity of each series. The isotherms correspond to type I according to IUPAC [22]. As observed, monolith samples named ACMCa2, ACMZn48 and ACMP48 (numbers “2” and “48” correspond to the percentage concentration of the activating agent used, as explained later) have the highest narrow micropore volume, lower than 0.7 nm, and are therefore those with the largest CO_2_ adsorption capacity in the experimental conditions studied. Sample ACMP48, and to a less extent sample ACMCa2, show a more open curvature at low relative pressures (*P*/*P*_0_ < 0.1), which indicates a wider pore size distribution, while sample ACMZn48 shows a steep curve with a more closed curvature in this pressure range, characteristic of highly microporous materials. Figure 1b shows the results of linear fitting to BET equation for samples ACMCa2, ACMP48, ACMZn48, relating *P*/*V(P*_0_*-P)* to *P*/*P*_0_, where *V* is the adsorbed volume. Correlation coefficients obtained (*R*_2_) for the three samples were 0.9999, 0.9995 and 0.9998, respectively.

Table 1 summarizes the textural properties of all samples, corresponding to the 12 carbon monoliths prepared, which have surface area values in the range 660–1700 m^2^·g^−1^ and pore volume values of 0.26–0.64 cm^3^·g^−1^. As observed, these parameters depend on the activating agent used, increasing in the order ACMZn < ACMP < ACMCa. The treatment with CaCl_2_ produces a stronger degradation of the precursor, so that after carbonization higher porosity and surface area are obtained for the monolith. In the case of samples ACMP and ACMZn, higher surface areas and pore volumes are reached with increasing concentrations of the activating agent solution. However, for samples treated with CaCl_2_ (ACMCa) the trend is the opposite, as observed in Table 1; this agent acts as a template in the developed porosity [23], after which with a washing process the agent is removed. With the increase of the Ca content in the carbonaceous materials a block is generated in the material, due to the increasing difficulty to remove the agent in the washing stage, causing a decrease in the textural characteristics of activated carbon. Likewise, it is possible that at high concentrations there is a sudden drop in both the surface and pore volume due to the partial retraction / destruction of the porous structure generated by the Ca content.

It was determined that the volume of narrow micropores by CO_2_ adsorption is between 0.23–0.43 cm^3^·g^−1^ for all monoliths; this value is more than 50% of the total microporosity, that which promotes the gas adsorption on the materials of interest. It is observed that with increasing surface area, the proportion of narrow microporosity increase, in each series, finding a maximum value of 0.43 cm^3^·g^−1^ on the MCa2 honeycomb which has the highest CO_2_ adsorption capacity.

### 2.2. Adsorption Isotherms of CO_2_

Carbon dioxide adsorption measurements were carried out on a volumetric adsorption apparatus (Quantachrome, Autosorb 3-B) at 273 K, up to a pressure of 1 Bar. In the adsorption tests, 100 mg of sample was used. Prior to adsorption, carbon materials were outgassed at a pressure of 1.31 × 10^−6^ atm for 3 h. Small amounts of CO_2_ were successively injected into the volumetric equipment and put in contact with the carbon sample, leaving enough time so that the adsorption equilibrium was reached for 3 min. The isotherms were measured in pressure between 0.1 and 1 Bar. Final pressure corresponding to equilibrium conditions was measured with highly accurate pressure sensors.

**Figure 2:**
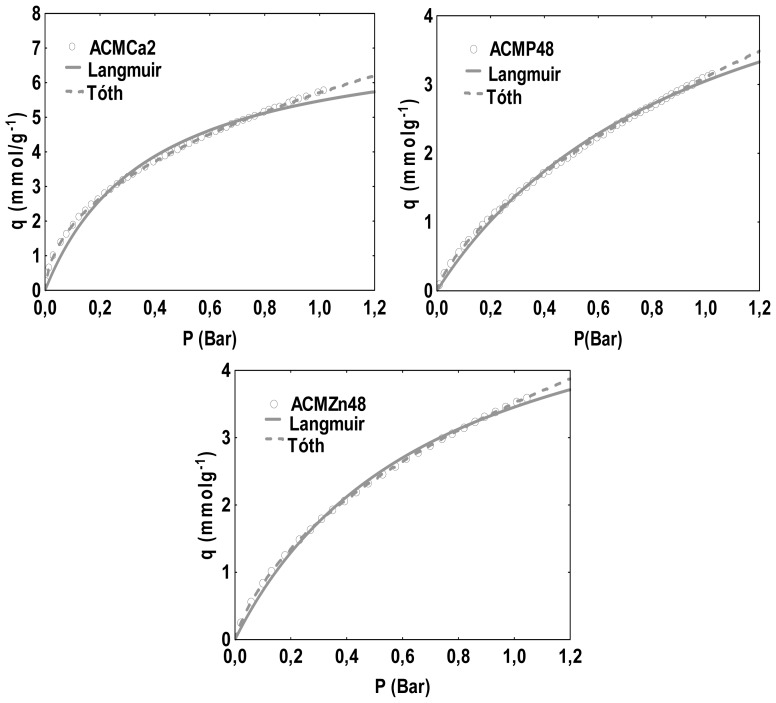
Adsorption isotherms of CO_2_ at 273 K of monolith samples ACMCa2, ACMZn48 and ACMP48 and curve fittings with Langmuir and Tóth equations.

Adsorption data were fitted to Langmuir and Tóth models by means of the STATISTICA^®^ program, calculating model parameters by the Quasi-Newton non linear regression method [24]. Figure 2 shows the experimental adsorption isotherms of CO_2_ at 273 K as well as the fitting curves to both models for carbon monolith samples ACMCa2, ACMZn48 and ACMP48. All isotherms correspond to type I of IUPAC classification, typical of microporous adsorbents. A good agreement is observed between experimental isotherms and both predicted curves for pressures up to 0.8 Bar, although the Tóth model shows a better fit in the whole range of pressures. For higher pressures the Langmuir equation seems to under-estimate the adsorption capacity, due to the deviation from the model hypothesis when pressure increases.

The fitting parameters to Langmuir and Tóth models are shown in Table 2. Values of *q_max_* for Langmuir equation are in the range 3643–7550 mmol·g^−1^, increasing with the concentration of H_3_PO_4_ and ZnCl_2_ solutions but decreasing with CaCl_2_ concentration with the exception of the ACMCa5 sample. On the other hand, values of Langmuir constant *K_L_*, which is related to the adsorption energy, are in the range 0.992–2.930 Bar^−1^, decreasing as the adsorption capacity of the adsorbents increases. The high values of correlation coefficients *R*^2^, ranging from 0.996 to 0.998, are evidence of the good agreement between experimental data and theoretical predictions.

In the case of the Tóth model, the parameter *q*_m_ of Equation 2 shows values in the range 1.008–3.608 mmol·g^−1^, as shown in Table 2, which are smaller than the values calculated with the Langmuir model, in the range 3.643–7.550 mmol·g^−1^. Experimental values for the highest pressure (2.627–5.756 mmol·g^−1^) were actually between the two predictions. This discrepancy can be due to the fact that both models are probably too simple to represent the real case of adsorption on the carbon materials of this study, and that according to Kinniburgh (1986) [25], prediction deviations even with a three-parameter model like the Tóth model, can be high when the amount of data fitted is not large enough. Values of *n* of Tóth model are in the range 0.457–0.707 (different than 1), confirming the surface heterogeneity of the carbon monoliths [18]. It can be emphasized that surface heterogeneity favors the adsorption of CO_2_, since the carbon monoliths that show the highest adsorption capacity, in their respective categories, not for the whole set of samples (ACMCa2, ACMZn48 and ACMP48) have also the lowest *n* values for each series of materials, and therefore the highest surface heterogeneity [18].

A maximum CO_2_ adsorption up to 5.75 mmolCO_2_·g^−1^ was obtained, the latter value is satisfactory, considering that there have been adsorbed amount of 5.22 mg and 4.09 mmolCO_2_·g^−1^, respectively on traditional 13X and 5A Zeolites [26,27], 3.68 mmolCO_2_·g^−1^, on sample PACM-28 obtained with the same precursor in our previous work [28], 3.34 mmolCO_2_·g^−1^ on Commercial granular activated carbon Norit CGP (N) [29], 1.34 mmolCO_2_·g^−1^ on sample N-PEHA which has amines incorporated through a wet impregnation method [29], and 8.36 mmolCO_2_·g^−1^, in carbon molecular sieves (VR-5-M) with a surface area of 3100 m^2^·g^−1^ and Vn above a 1.40 cm^3^·g^−1^ [30]; all these adsorption capacities under the same conditions of pressure.

## 3. Experimental Section

**Figure 3:**
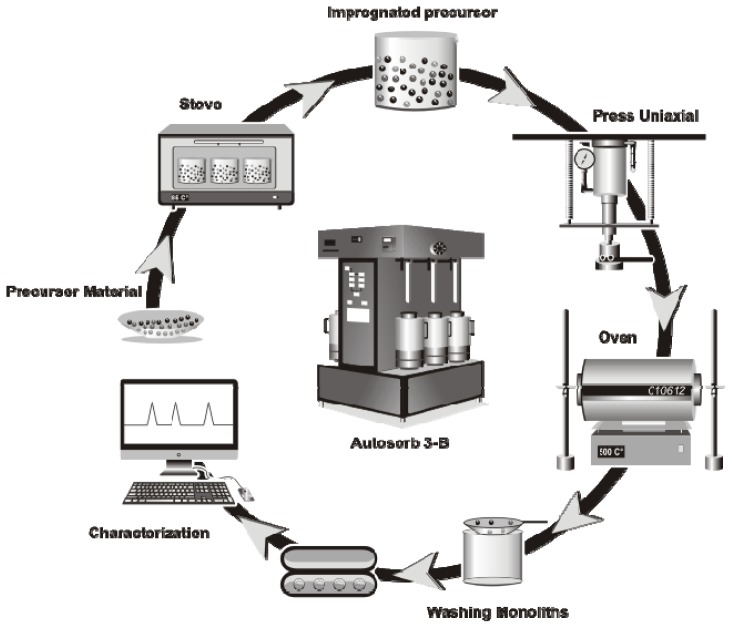
Scheme of the preparation process of the activated carbon monoliths.

**Figure 4:**
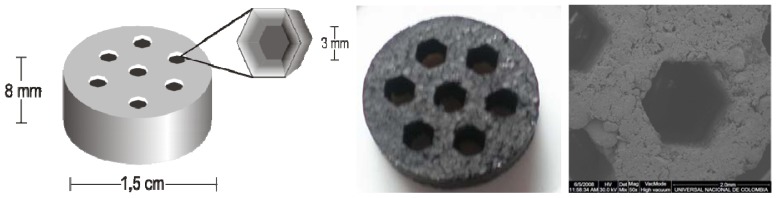
Geometric parameters of the compact activated carbon monoliths obtained. The scheme (**left**), a picture (**center**) and microphotography SEM (**right**) of the activated carbon monolith.

The activated carbon monoliths were prepared using raw African palm shells as a lignocellulosic precursor. The impregnation step was carried out with a ratio of 2 mL of solution/1 g material precursor, by using either 32%–48% H_3_PO_4_ aqueous solutions (samples named ACMP), 32%–48% ZnCl_2_ solutions (samples named ACMZn) or 2%–5% CaCl_2_ solutions (samples named ACMCa). The particle size of the precursor solid was initially reduced to 38 μm, for which the material was washed, triturated with a commercial mill and subsequently sieved and then impregnated. These materials were subjected to a pressure of 6500 psi in a uniaxial press apparatus, at a temperature of 423 K. The resulting monoliths were cylindrical, having an approximate size of 1.5 cm external diameter and 8 mm height, with a series of six hexagonal parallel channels of about 3 mm diameter each. Figure 3 shows a scheme of the preparation process of the monoliths. Figure 4 presents the scheme, a picture and microphotography SEM of the activated carbon monolith obtained, in these the morphology of the monoliths that have seven transverse channels with the dimensions shown below can be seen. The hexagonal morphology was chosen for the monoliths due to the fact that hexagonal channel monoliths present higher internal and external mass transfer coefficients and lower pressure drops than square ones [31].

The textural properties of the synthesized monoliths were analyzed by using N_2_ adsorption at 77 K in volumetric adsorption equipment (Quantachrome, Autosorb 3-B). For these isotherms, 100 mg of sample was used, before adsorption the samples were outgassed at 523 K for 24 h. The micropore volume, *V*_0_ (N_2_), was obtained by applying the Dubinin-Radushkevich equation to the nitrogen adsorption data. The total pore volume, *V_t_*, was obtained from the amount adsorbed at a relative pressure *P/P_0_* of 0.99, while the mesopore volume, *V_meso_*, was obtained by the difference between the total pore volume and the micropore volume [19].

## 4. Conclusions

The results of carbon dioxide adsorption at 273 K on three series of activated carbon monoliths prepared by impregnation of African palm shells with H_3_PO_4_, ZnCl_2_ and CaCl_2_ aqueous solutions show that these materials can be interesting for CO_2_ capture. The experimental data of adsorption can be fitted to Langmuir and Tóth models, with correlation coefficients of 0.998 and 0.999, respectively. Surface area values obtained are as high as 1700 m^2^·g^−1^, and total pore volumes up to 0.64 cm^3^·g^−1^, corresponding to the carbon monolith sample which was activated with calcium chloride (ACMCa2). Data fitting to the theoretical models allows to conclude that carbon dioxide adsorption on the carbon monoliths is related to the surface heterogeneity of the adsorbent materials, the data better reproduced by Tóth model. The carbon monoliths obtained can adsorb as much CO_2_ as 5.8 mmolCO_2_ g^−1^ at 1 Bar and 273 K.

## Figures and Tables

**Table 1:** Textural parameters for activated carbon monoliths, obtained from N_2_ adsorption isotherms at 77 K and CO_2_ adsorption isotherms at 273 K.

N_2_ adsorption 77 K					CO_2_ adsorption 273 K
	
Sample	S_BET_ (m^2^·g^−1^)	*V*_O_^a^ (cm^3^·g^−1^)	*V*_meso_^b^ (cm^3^·g^−1^)	*V*_0.99_^c^ (cm^3^·g^−1^)	*V*_n_^d^ (cm^3^·g^−1^)
ACMP32	1020	0.40	0.09	0.48	0.25
ACMP36	1057	0.40	0.10	0.50	0.27
ACMP40	1078	0.41	0.11	0.52	0.28
ACMP48	1368	0.48	0.18	0.66	0.32
ACMZn32	660	0.26	0.03	0.29	0.23
ACMZn36	845	0.34	0.02	0.36	0.34
ACMZn40	884	0.35	0.03	0.38	0.34
ACMZn48	924	0.37	0.01	0.38	0.36
ACMCa2	1700	0.64	0.10	0.74	0.43
ACMCa3	1469	0.60	0.04	0.64	0.34
ACMCa5	1445	0.57	0.06	0.63	0.36
ACMCa7	926	0.37	0.05	0.42	0.23

a Micropore Volume (N_2_);

b Mesopore Volume (N_2_);

c Total Volume (N_2_);

d Narrow micropore Volume (CO_2_).

**Table 2:** Parameters of Langmuir and Tóth models for CO_2_ adsorption.

Sample	Langmuir			Tóth			
	
	*q*_max_ (mmol·g^−1^)	*K*_L_ (Bar^−1^)	*R*^2^	*q*_m_ (mmol·g^−1^)	*α_T_* (Bar^−1^)	*n*	*R*^2^
ACMP32	4.609	1.529	0.996	1.008	44.60	0.519	0.999
ACMP36	4.757	1.464	0.996	1.068	44.17	0.556	0.999
ACMP40	4.854	1.391	0.997	1.402	25.34	0.527	0.999
ACMP48	5.122	0.992	0.998	1.592	21.70	0.457	0.999
ACMZn32	3.786	2.068	0.998	2.398	8.417	0.687	0.999
ACMZn36	5.606	1.928	0.998	3.608	7.658	0.677	0.999
ACMZn40	5.687	1.829	0.998	3.523	7.967	0.658	0.999
ACMZn48	5.939	1.390	0.998	3.211	8.332	0.593	1.000
ACMCa2	7.550	2.638	0.996	1.116	185.4	0.570	0.999
ACMCa3	3.807	2.770	0.996	1.762	32.26	0.707	0.999
ACMCa5	5.709	2.870	0.997	2.498	24.68	0.663	0.999
ACMCa7	3.643	2.930	0.998	1.535	27.24	0.659	0.999
